# Latent and active *ab*PPO4 mushroom tyrosinase cocrystallized with hexatungstotellurate(VI) in a single crystal

**DOI:** 10.1107/S1399004714013777

**Published:** 2014-08-29

**Authors:** Stephan Gerhard Mauracher, Christian Molitor, Rami Al-Oweini, Ulrich Kortz, Annette Rompel

**Affiliations:** a Institut für Biophysikalische Chemie, Fakultät für Chemie, Universität Wien, Althanstrasse 14, 1090 Wien, Austria; b School of Engineering and Science, Jacobs University, PO Box 750 561, 28725 Bremen, Germany

**Keywords:** *ab*PPO4, tyrosinase, polyphenol oxidase, *Agaricus bisporus*

## Abstract

Tyrosinases, bifunctional metalloenzymes, catalyze the oxidation of monophenols and *o*-diphenols to *o*-quinones, the precursor compounds of the brown-coloured pigment melanin. In eukaryotic organisms, tyrosinases are expressed as latent zymogens that have to be proteolytically cleaved in order to form highly active enzymes. This activation mechanism, known as the tyrosinase maturation process, has scientific and industrial significance with respect to biochemical and technical applications of the enzyme. Here, not only the first crystal structure of the mushroom tyrosinase *ab*PPO4 is presented in its active form (Ser2–Ser383) and in its 21 kDa heavier latent form (Ser2–Thr545), but furthermore the simultaneous presence of both forms within one single-crystal structure is shown. This allows for a simple approach to investigate the transition between these two forms. Isoform *ab*PPO4 was isolated and extensively purified from the natural source (*Agaricus bisporus*), which contains a total of six polyphenol oxidases (PPOs). The enzyme formed crystals (diffracting to a resolution of 2.76 Å) owing to the employment of the 6-tungstotellurate(VI) salt (Na_6_[TeW_6_O_24_]·22H_2_O) as a cocrystallization agent. Two of these disc-shaped Anderson-type polyoxoanions [TeW_6_O_24_]^6−^ separate two asymmetric units comprising one crystallographic heterodimer of *ab*PPO4, thus resulting in very interesting crystal packing.

## Abstract 2

Mushroom tyrosinase isoform *ab*PPO4 (*Agaricus bisporus* polyphenol oxidase 4) was crystallized by means of an Anderson-type polyoxometalate. The enzyme crystallized as a crystallographic heterodimer containing the zymogen (L-TYR; 64 kDa), the 21 kDa smaller activated form (A-TYR) and the polyoxometalate (POM) within one single crystal in a 1:1:1 ratio.

## 1. Introduction   

Tyrosinase (TYR) is a representative of the type-3 copper protein family capable of catalyzing two chemical reactions responsible for the formation of melanin. These reactions, the oxidation of monophenols to *ortho*-quinones (monophenolase activity EC 1.14.18.1 and diphenolase activity EC 1.10.3.1) and the oxidation of *ortho*-diphenols to the respective *o*-quinones (diphenolase activity EC 1.10.3.1), are solely catalyzed by tyrosinases (Ramsden & Riley, 2014 ▶). Other representatives of this protein family, namely catechol oxidases, are deficient in monophenolase activity. Haemocyanins lack both activities but act as oxygen-transport proteins. Tyrosinases and catechol oxidases are generally classified as polyphenol oxidases (PPOs), which represent an area of research that has led to a considerable number of reports in the past 120 years (Bourquelot & Bertrand, 1895 ▶; Seo *et al.*, 2003 ▶; Solomon *et al.*, 2014 ▶). In particular, tyrosinase enzyme kinetics, the browning of agricultural products (Jolivet *et al.*, 1998 ▶), applications in organic syntheses (Durán *et al.*, 2002 ▶), wastewater regeneration (Karam & Nicell, 1997 ▶; Xu & Yang, 2013 ▶), protein cross-linking (Thalmann & Lötzbeyer, 2002 ▶; Jus *et al.*, 2011 ▶), medical interest in human TYR (Fitzpatrick, 1952 ▶; Vijayan *et al.*, 1982 ▶; Spritz *et al.*, 1990 ▶; Tripathi *et al.*, 1991 ▶) and structural studies have been among other topics of interest.

Eukaryotic tyrosinases are expressed as inactive zymogens (∼50–80 kDa; latent L-TYR) before being proteolytically activated by the removal of a C-terminal active-site-shielding domain (∼20 kDa) (Faccio *et al.*, 2013 ▶; Fujieda *et al.*, 2013 ▶). This activation process is commonly known as the tyrosinase-activation/maturation process and has recently been extensively investigated. Evidently, the function of the C-terminal domain is not restricted to a shutter function but it may also act as molecular machinery to incorporate copper ions into the active site (Fujieda *et al.*, 2013 ▶). Owing to the cell toxicity (Gaetke & Chow, 2003 ▶) of free copper ions, such protein-assisted metal-incorporation mechanisms by chaperones (Robinson & Winge, 2010 ▶) are commonly known and have been described for the cocrystallized caddie protein of a bacterial tyrosinase (Matoba *et al.*, 2006 ▶, 2011 ▶).

To date, the structures of two recombinantly expressed bacterial tyrosinases [from *Streptomyces castaneoglobisporus* (*sc*TYR; PDB entry 2ahk; Matoba *et al.*, 2006 ▶) and *Bacillus megaterium* (PDB entry 3nm8; Sendovski *et al.*, 2011 ▶)], one recombinantly expressed fungal pro-tyrosinase (L-TYR from *Aspergillus oryzae*; *ao*TYR; PDB entry 3w6w; Fujieda *et al.*, 2013 ▶) and of the proteolytically activated (A-TYR) fungal tyrosinase of isoform *ab*PPO3 (*Agaricus bisporus*; PDB entry 2y9w; Ismaya *et al.*, 2011 ▶) have been published. Moreover, protein structures related to tyrosinases are those of catechol oxidases from plants and fungi [*Ipomoea batatas* (*ib*CO; PDB entry 1bt3; Klabunde *et al.*, 1998 ▶), *Vitis vinifera* (PDB entry 2p3x; Virador *et al.*, 2009 ▶) and *Aspergillus oryzae* (L-PPO, PDB entry 4j3p; A-PPO, PDB entry 4j3q; Hakulinen *et al.*, 2013 ▶)], several haemocyanin structures (including those from *Megathura crenulata* (PDB entry 3qjo; Jaenicke *et al.*, 2011 ▶) and *Enteroctopus dofleini* (PDB entry 1js8; Cuff *et al.*, 1998 ▶)] and two arthropodal prophenoloxidases [from *Manduca sexta*, PDB ID: 3hhs (Li *et al.*, 2009 ▶); and from *Marsupenaeus, japonicus*, PDB ID: 3wky (Masuda *et al.*, 2014 ▶)].

Today, six tyrosinase isoforms (PPO1–6) originating from six genes located on two different (from a total of 12) chromosomes (PPO1 and 6 on chromosome 8; PPO2–5 on chromosome 12) of the common edible mushroom (*A. bisporus*) are known (Wichers *et al.*, 2003 ▶; Wu *et al.*, 2010 ▶; Weijn *et al.*, 2013 ▶). Apart from differing abundances with respect to the tissue compartment and the fruiting-body growth stage (Hammond & Nichols, 1976 ▶), no unique function of a particular isoform has been published (Li *et al.*, 2011 ▶; Weijn *et al.*, 2013 ▶).

Polyoxometalates (POMs), which are anionic metal-oxo clusters of early transition metals in high oxidation states, exhibit a unique structural and compositional variety (Pope, 1983 ▶; Pope & Kortz, 2012 ▶). POMs have interesting electronic, redox, catalytic, optical, thermal, magnetic and bioactive properties and are hence studied in many different fields of science and technology (Müller *et al.*, 2001 ▶; Schnack *et al.*, 2006 ▶; Kortz *et al.*, 2009 ▶; Iqbal *et al.*, 2013 ▶; Jahier *et al.*, 2013 ▶). The high negative charge, large size, shape and water-solubility of POMs allows binding to proteins, which enables their utilization as effective protein-crystallization agents (Zhang *et al.*, 2007 ▶). However, the employment of POMs in this regard has so far mostly been limited to crystal-soaking experiments, exploiting their potential for phasing by either isomorphous replacement or anomalous scattering (Corey *et al.*, 1962 ▶; Ladenstein *et al.*, 1987 ▶; O’Halloran *et al.*, 1987 ▶; Thygesen *et al.*, 1996 ▶; Rudenko *et al.*, 2003 ▶; Zebisch *et al.*, 2012 ▶; Dahms *et al.*, 2013 ▶). Additionally, POMs have been implemented as contrast agents in protein crystallography in order to elucidate buried compound channels in larger proteins (Dauter, 2005 ▶). It is also worth noting the famous crystal structure of the 50S ribosome (*Deinococcus radiodurans*) where crystals were soaked with the Keggin-type POM salt K_5_H[PW_12_O_40_]·12H_2_O (Schluenzen *et al.*, 2000 ▶; Harms *et al.*, 2001 ▶; Pioletti *et al.*, 2001 ▶).

Based on X-ray crystallographic studies, POMs (mostly of the Keggin, Lindqvist and Well–Dawson types) are part of the structure of around 25 PDB entries (Pioletti *et al.*, 2001 ▶; Locher *et al.*, 2002 ▶; Kowalewski *et al.*, 2012 ▶; Zebisch *et al.*, 2012 ▶, 2014 ▶). However, owing to mostly poorly defined electron densities at POM binding locations described in the literature or to a lack of interest in binding modes because POMs were only incorporated for phasing purposes, a detailed structural POM binding mode to proteins has so far rarely been defined. The exceptions are a self-assembled POM (PDB entries 4f6t and 2ogx) in a molybdenum-storage protein (Schemberg *et al.*, 2007 ▶) and a very recent structure of NTPDase1 (PDB entries 4bvo, 4bvp and 4brh; Zebisch *et al.*, 2014 ▶). The latter publication describes in detail the binding modes of soaked and cocrystallized POMs, namely dodecatungstate, decavanadate and octamolybdate/hepta­molybdate. Interestingly, besides the predominantly electrostatic interactions and hydrogen bonds described in this study, covalently bonded POMs have also been identified (Zebisch *et al.*, 2014 ▶).

The mushroom tyrosinase *ab*PPO4 was extensively purified and crystallized using a method described elsewhere (Mauracher, Molitor, Al-Oweini *et al.*, 2014 ▶; Mauracher, Molitor, Michael *et al.*, 2014 ▶). In the present crystallographic study, the crystal structure of active *ab*PPO4 (UniProt C7FF05 and K9I869) and its zymogen, simultaneously present in one structure, are described. Hence, the enzyme cocrystallized in its active (A-TYR) and latent (L-TYR) forms as a crystallo­graphic heterodimer within the same asymmetric unit. The origin of A-TYR in the crystallization batch is unclear since to all intents and purposes only L-TYR was used to set up the crystallization trials. However, minor protease impurities combined with long storage times of the purified protein solution resulting in partial activation may offer an explanation. Owing to the peculiar heteromeric structure, a direct comparison between A-TYR and L-TYR is feasible and allows new insights into the tyrosinase-maturation process. The sodium salt of 6-tungstotellurate(VI) (Na_6_[TeW_6_O_24_]·22H_2_O; PDB ligand ID TEW) was crucial for the formation of single crystals and showed an exceptional assembly in this crystal structure. The polyoxoanion [TeW_6_O_24_]^6−^ with the Anderson–Evans-type structure was deliberately used as a cocrystallization agent to obtain single crystals suitable for X-ray diffraction. Hence, a detailed description of its binding mode and high-order arrangement is presented herein.

## 2. Materials and methods   

### 2.1. Protein purification and crystallization   

Protein purification and crystallization was performed as described elsewhere (Mauracher, Molitor, Al-Oweini *et al.*, 2014 ▶; Mauracher, Molitor, Michael *et al.*, 2014 ▶). Briefly, the protein was extensively purified from the natural source (*A. bisporus* fruiting-body stipes) by an extraction method including detergent and aqueous polymer phase separations followed by a subsequent multi-step chromatographic purification procedure *via* FPLC (fast protein liquid chromatography). Protein purity was checked by SDS–PAGE and mass spectrometry (nano-ESI QTOF). The protein was crystallized by means of the hanging-drop vapour-diffusion technique with a protein concentration of 10 µg µl^−1^, a drop volume of 1 µl (0.5 µl protein solution and 0.5 µl reservoir solution) and a reservoir volume of 500 µl. The crystallization conditions were 10% PEG 4000, 1 m*M* Na_6_[TeW_6_O_24_]·22H_2_O, 25 m*M* Tris–HCl pH 7.5 at 291 K. Crystals grew within 1–5 d to approximate dimensions of 300 × 30 × 10 µm.

### 2.2. Data collection and processing   

Data collection was performed as described elsewhere (Mauracher, Molitor, Al-Oweini *et al.*, 2014 ▶) at Diamond Light Source (DLS), Oxfordshire, England on the monochromatic (0.9173 Å) MX beamline I04-1. The obtained diffraction data sets were processed with the *XDS* program package (version March 30, 2013; Kabsch, 2010 ▶). Data-collection statistics are given in Table 1 ▶.

### 2.3. Structure solution and refinement   

Initial phase determinations using molecular replacement (MR) with *Phaser* from the *PHENIX* program suite (Adams *et al.*, 2010 ▶) were performed using a model of the *ab*PPO3 mushroom tyrosinase structure (PDB entry 2y9w, chain *H*; Ismaya *et al.*, 2011 ▶) modified with the sequence of *ab*PPO4 (UniProt K9I869) derived from a comparative protein structure modelling program (*MODELLER*; Šali & Blundell, 1993 ▶). As predicted from the Matthews coefficient (*phenix.xtriage*; 46% solvent content), two molecules fitted in the asymmetric subunit. Notably, in the initial electron-density maps, two highly dense disc-shaped sites were visible, corresponding to the shape of the cocrystallized polyoxoanion [TeW_6_O_24_]^6−^. The rotated and translated model was employed to perform MR/SAD phasing applying *AutoSol* from the *PHENIX* program suite. This phase-determination operation was possible owing to the anomalous signal of the cocrystallized POM anion [TeW_6_O_24_]^6−^. The corresponding *f*′ and *f*′′ values of tungsten at the corresponding wavelength (0.92 Å) are −3.79 and 10.42 e, respectively. The electron density thus generated was used for initial model building. Moreover, the two polyoxoanions were incorporated into the model by placing the structural data of the respective POM and the corresponding restraint data in the PDB file (generated by *phenix.reel* and *phenix.elbow* from the crystal structure of TEW; Schmidt *et al.*, 1986 ▶). Notably, owing to the positioning of the POMs on a twofold axis their occupancy was set to 50%.

Based on the lack of structural information for the C-terminal domain, the respective model ended with residue Gly379. No electron density was visible extending beyond residue Gly379 of chain *A*; however, this was not the case in chain *B*. Several tube-shaped electron-density convolutions were visible in the near-vicinity of Gly379 in a volume of approximately 10.000 Å^3^. Applying the automated model-building and refinement program *AutoBuild* from the *PHENIX* program suite indicated two β-strands corresponding to the sequence sections Phe428–Tyr447 and Phe482–Tyr498. However, no further reasonable solution was generated using automated modelling programs for the missing residues of the C-terminal domain of chain *B*. Therefore, we completed the missing chain by placing alanine residues in the vacant electron density and applying intermediate refinement steps (*phenix.refine* v.1.8.4_1496 from the *PHENIX* program suite and *REFMAC* from the *CCP*4 program suite; Murshudov *et al.*, 2011 ▶; Winn *et al.*, 2011 ▶). The alanine residues were then successively replaced by the respective amino-acid residues derived from the sequence (UniProt K9I869). While improving the model *R* values, we repeated phase determination by *AutoSol* (MR/SAD) with the respective optimized model. This was performed until all of the statistical parameters of the model were in an adequate range for deposition of the structure in the PDB.

### 2.4. Model validation and deposition   

Structural models were assessed with respect to the experimental data using *SFCHECK* (Vaguine *et al.*, 1999 ▶). Geometric parameters were validated by *Coot* (Emsley *et al.*, 2010 ▶) and *MolProbity* (Chen *et al.*, 2010 ▶). The model was deposited in the PDB (http://www.rcsb.org) as entry 4oua.

## 3. Results and discussion   

### 3.1. Overall structure of the asymmetric unit of mushroom tyrosinase *ab*PPO4   

**Figure 1:**
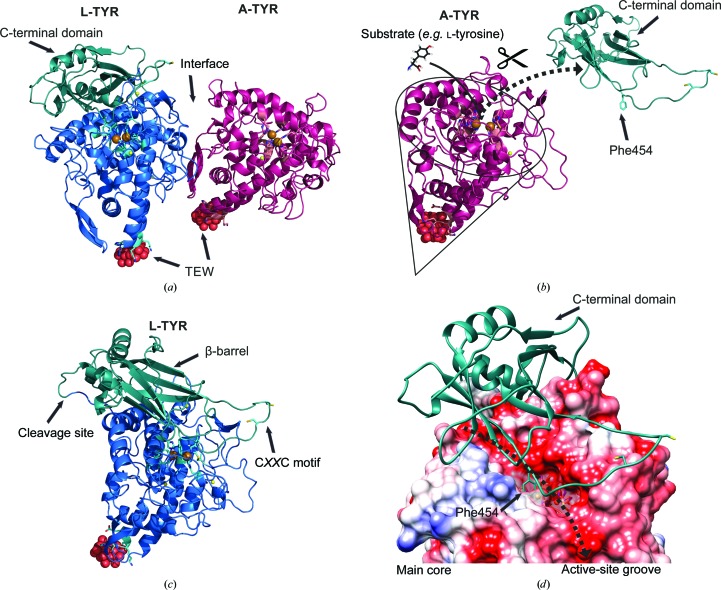
Overall structure. Colour code: L-TYR main core, blue; C-terminal domain, turquoise; A-TYR, purple; sodium ions, yellow; copper ions, bronze; POM (TEW) oxygen, red; tellurium, grey; positive electrostatic potential of coulombic surface, blue; negative electrostatic potential of coulombic surface, red. (*a*) Overall structure of asymmetric unit; the crystallographic heterodimer of *ab*PPO4. L-TYR is shown on the left and A-TYR is depicted on the right. (*b*) Illustration of the activation process. By the proteolytic removal of the C-terminal domain the active site becomes solvent-exposed and substrates (*e.g.* l-tyrosine) are able to approach. (*c*) Different viewing angle of the single monomer of L-TYR to illustrate the β-barrel-shaped C-terminal domain, the proteolytic cleavage site and the putative C*XX*C motif-containing copper grappler. (*d*) Coulombic surface illustration of the L-TYR main core with the C-terminal domain (cartoon) attached. The solvent-accessible groove, exhibiting a strong negative electrostatic potential, is indicated by the dashed arrow. Phe454 is shown to protrude into the active site (transparent surface around the active site).

The crystal structure of mushroom tyrosinase *ab*PPO4 was solved by combining molecular replacement (MR) and single-wavelength anomalous dispersion (SAD), benefiting from the anomalous scattering signal of the tungsten provided by the cocrystallized POM. In Fig. 1 ▶(*a*) an overall view of the asymmetric unit of *ab*PPO4 is shown. The protein crystallized in space group *C*2 as a crystallo­graphic heterodimer composed of one polypeptide chain of L-TYR (Ser2–Thr556) and one chain of A-TYR (Ser2–Ser383) as well as two half POM discs ([TeW_6_O_24_]^6−^). Both POMs lie on a crystallographic twofold axis and show an occupancy of 1.00 generated by two superimposing POM disks (occupancy of 0.5) originating from two oppositely positioned asymmetric units. The final structure was refined to a resolution of 2.76 Å. The complete crystallographic data set is described in Table 2 ▶.

### 3.2. Overall structure of *ab*PPO4 A-TYR   

**Figure 2:**
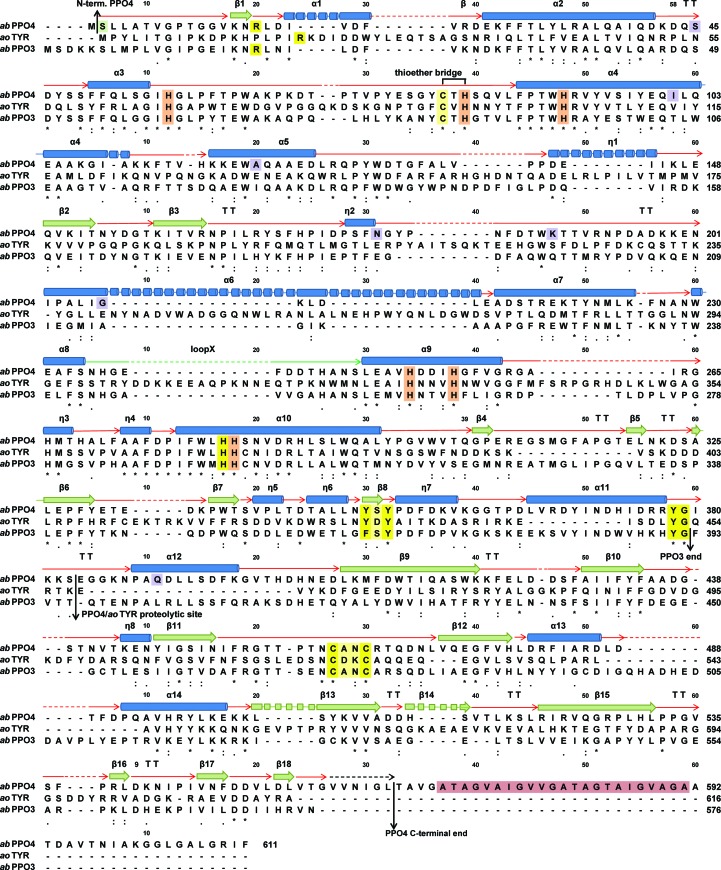
Multiple sequence alignment of *ab*PPO4 (UniProt K9I869), *ao*TYR (UniProt B8NJ95) and *ab*PPO3 (UniProt C7FF04). Colour code for secondary-structure elements: α-helices, blue tubes; β-strands, green arrows; loops, red arrows; hydrogen-bonded turnovers, TT. Colour code for highlighted residues: copper-binding histidines, orange; conserved tyrosinase motifs, yellow; mutations discerning *ab*PPO4 K9I869 from *ab*PPO4 C7FF04, purple; putative transmembrane anchor (Ala569–Ala591), raspberry; acetylated N-terminus, green.

The A-TYR chain lacking the C-terminal domain (Ser2–Ser383) has an approximately conical shape tapered at the POM binding site, with a basal diameter of ∼50 Å and a height of ∼40 Å (Fig. 1 ▶ *b*). The secondary-structure elements are identified and are numbered sequentially (α for α-helix, η for 3_10_-helix, β for β-strand and TT for hydrogen-bonded turn; Fig. 2 ▶). The A-TYR chain consists of 382 continuous amino acids ending with a cleavage site after Ser383 as determined by mass spectrometry (MS; Mauracher, Molitor, Michael *et al.*, 2014 ▶). There are a number of hydrogen bonds present that are not part of the secondary-structure elements. These hydrogen bonds connect the turnover region α4/α5 to the β-bundle β2/β3 (Thr114 N to Tyr155 O, *d* = 2.7 Å) and the N-terminal end to loop Ile56–Leu86 (Leu3 N to Ala65 O, *d* = 2.8 Å), and several others are importantly involved in defining the structure (Arg16 N to Ser351 O, *d* = 2.8 Å; Thr190 N to Tyr169 O, *d* = 3.0 Å; Gly78 O to Met312 N, *d* = 2.6 Å; Ile19 N to Tyr131 O, *d* = 3.0 Å; Trp132 N to Leu281 O, *d* = 2.8 Å). In addition to the type-3 copper centre, another metal-binding site, occupied by sodium, is also present (Na1A; Gly55 O, Gly58 O, Pro60 O and H_2_O126/127; Figs. 1*b* and 4*b*).

#### 3.2.1. Overall structure of *ab*PPO4 L-TYR   

The main core of L-TYR (Ser2–Ser383; Fig. 1 ▶ *c*) exhibits a very similar shape to that of A-TYR (Fig. 1 ▶ *b*). It has the same conical shape but has the C-terminal domain (Glu384–Gly558) placed on the ‘flat bottom’ side with approximate dimensions of 50 × 40 × 18 Å. A total of 555 continuous amino acids could be assigned to the respective well defined electron density for the L-TYR chain. Only the small disordered C-terminal ending loop is missing (Val559–Thr565). This terminal region has a ragged constitution in that the C-terminal amino acid differs amongst the variants, as demonstrated by MS measurements (Mauracher, Molitor, Michael *et al.*, 2014 ▶). The secondary-structure elements on the L-TYR chain are identified and are numbered sequentially (α for α-helix, η for 3_10_-helix, π for π-helix, β for β-strand and TT for hydrogen-bonded turn; Fig. 2 ▶). Apart from the same hydrogen bonds as indicated in the main core of A-TYR, three hydrogen bonds between the L-TYR main core and the C-terminal domain are present (Ile380 N to Lys398 O, *d* = 2.7 Å; Ala270 N to Arg455 O, *d* = 2.9 Å; Glu238 N to Val472 O, *d* = 2.9 Å). In addition to the type-3 copper centre, three metal-binding sites, occupied by sodium, were found (Na1A, Gly55 O, Gly58 O, Pro60 O and H_2_O126/127; Na2B, Gly237 O, Asn235 O and Glu331 O; Na3B, Leu348 O, Glu99 O^∊1^, Arg16 N^η2^ and Ser96 O^γ^) (Fig. 1 ▶ *c*).

#### 3.2.2. Crystal-packing interfaces in A-TYR and L-TYR   

*PISA* (*Proteins, Interfaces, Structures and Assemblies*; Krissinel & Henrick, 2007 ▶) studies have shown that an interface between A-TYR and L-TYR in the asymmetric unit has no probability of formation (complex-formation significance score of 0.00) in solution (Fig. 1 ▶ *a*). No strong interactions were predicted for an interface area of 526.6 Å^2^ (surface area: A-TYR, 15 388 Å^2^; L-TYR, 21 827 Å^2^) with a Δ*G* value of −0.9 kcal mol^−1^ and a Δ*G* *P*-value of 0.67. However, one hydrogen bond (*A* Gln149 N^∊2^ to *B* Thr343 O^γ1^, *d* = 3.21 Å) as well as three salt bridges (*A* Asp41 O^δ1^ to *B* Lys335 N^ζ^, *d* = 3.8 Å; *A* Arg163 N^η2^ to *B* Glu331 O^∊1^, *d* = 2.51 Å; *A* Arg163 N^η2^ to *B* Glu331 O^∊2^, *d* = 3.76 Å) are present. In addition to the asymmetric unit interface, six other reasonable interfaces in the crystal packing between protein surfaces, none of which display any reasonable interaction for forming quaternary structures in solution, are described by *PISA* calculations. Thus, it is assumed that both A-TYR *ab*PPO4 and L-TYR *ab*PPO4 exist as monomers in solution.

**Figure 3:**
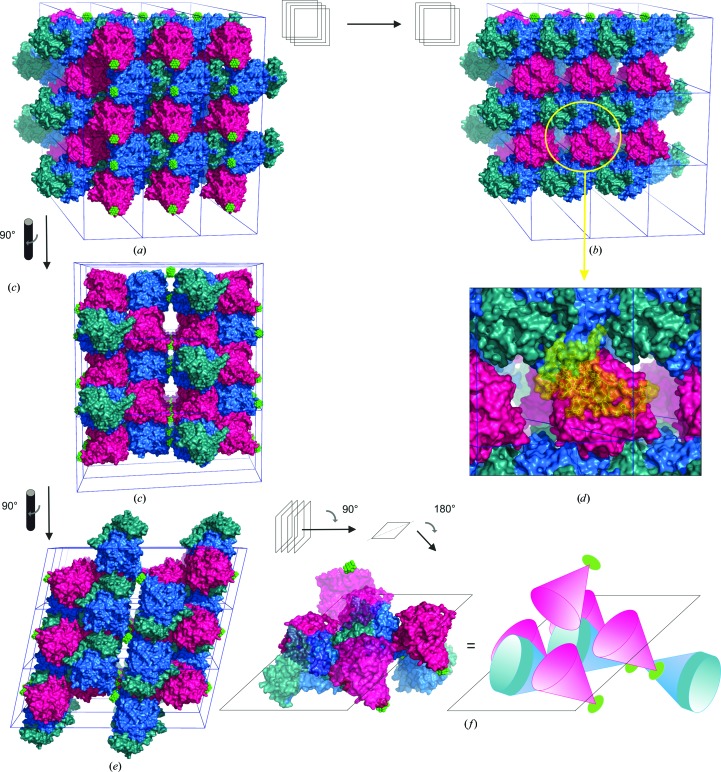
Crystal packing. Colour code: L-TYR main core, blue; C-terminal domain, turquoise; A-TYR, purple; POM (TEW), green. (*a*) Crystal packing in 1 × 3 × 3 supercell. Four layers of protein (surface illustration) pointing alternately in opposing directions. The front layer exposes its POM side (front). (*b*) The same view as in (*a*) but with the first layer removed. Hence, the protein side is exposed (back). Here, the head-to-body alignment of L-TYR is discernible. (*c*) The image in (*a*) rotated by 90°, showing the sandwich-like layer stacking. (*d*) The insert shows a C-terminal domain attached notionally to A-TYR and clearly clashing with L-TYR of the adjacent asymmetric unit. (*e*) The image in (*c*) rotated by 90°. (*f*) The image in (*e*) tilted by 90° and rotated by 180°. One central L-TYR chain surrounded by the vicinal flanking chains (5× A-TYR, 2× L-TYR). An equivalent schematic illustration is shown on the right.

The crystal exhibits a relatively high solvent content of 55.2% (Matthews, 1968 ▶; *V*_M_ = 2.74 Å^3^ Da^−1^). Fig. 3 ▶ illustrates the crystal packing in a 1 × 3 × 3 supercell (nine unit cells).

The packing is best described as a stack of protein layers by defining the front surface of the layer as the POM-exposed side (Fig. 3 ▶ *a*) and the back surface as the protein-exposed side (Fig. 3 ▶ *b*). Consequently, two layers are stacked together in a back-to-back fashion oriented in opposing directions. Such a double layer is further stacked *via* its front side (protein–POM ‘sandwich’) with another double layer orientated in equivalent directions (Figs. 3 ▶ *c* and 3 ▶ *d*).

The protein layers are composed in such a way that when an L-TYR chain is viewed centrally and its C-terminal domain is considered to be the head then L-TYR is positioned uni­directionally (head to body; Figs. 3 ▶ *b* and 3 ▶ *e*). Furthermore, L-TYR is surrounded by five A-TYR chains, of which two are bilaterally positioned at the head (C-terminal domain), two are bilaterally positioned at the body (main core) and one is positioned atop the head (Fig. 3 ▶ *e*).

Interestingly, the C-terminal domain of L-TYR is crucially involved in crystal packing. The main cores of A-TYR and L-TYR are not positioned equally. Moreover, as illustrated in Fig. 3 ▶(*d*), a hypothetical attachment of a C-terminal domain to A-TYR would result in a massive clash with the L-TYR main core of another asymmetric unit. Hence, a similar crystal packing for a crystal composed exclusively of L-TYR can be excluded and the present packing is only feasible owing to the heterogeneity provided by the presence of both forms.

#### 3.2.3. Superimposition of A-TYR and L-TYR   

**Figure 4:**
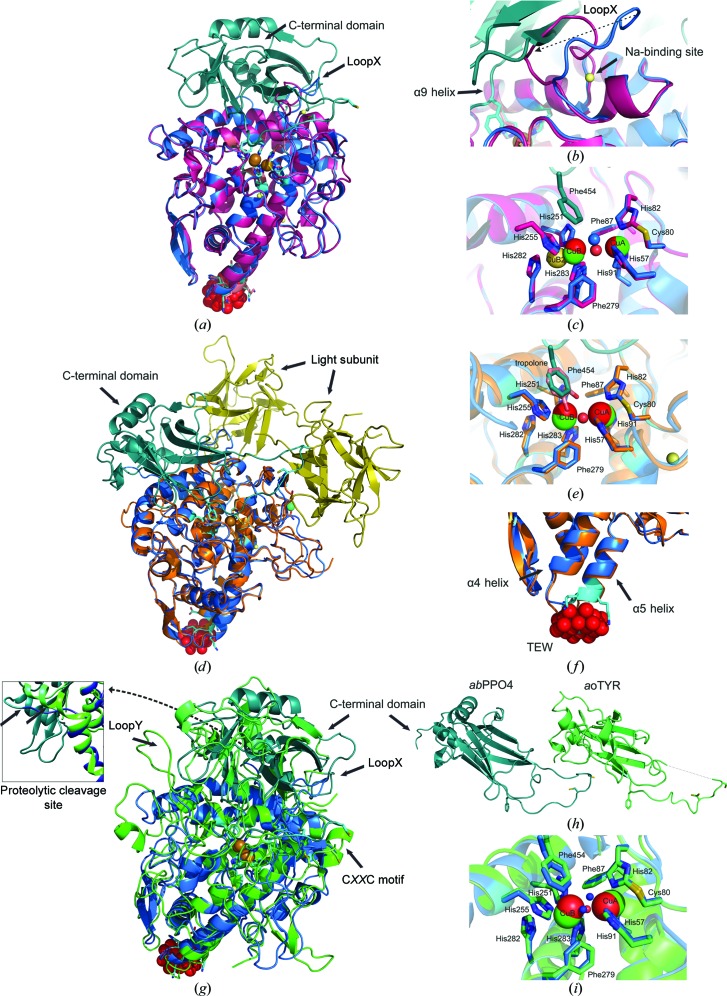
Superimpositions. Colour code: L-TYR main core, blue; L-TYR C-terminal domain, turquoise; A-TYR main core, purple; *ao*TYR main core, green; *ao*TYR C-terminal domain, lime green; *ab*PPO3 main core, orange; *ab*PPO3 light subunit, yellow; L-TYR *ab*PPO4 copper ions, green; respective superimposed copper ions, red; sodium ions, yellow; water molecules, blue; tropolone, pink; POM oxygen spheres, red. (*a*) Superimposition of L-TYR *ab*PPO4 with A-TYR *ab*PPO4. (*b*) Magnified region of loopX (Asn236–Ser246) which is pushed sideways by the attachment of the C-terminal domain. The dashed arrow indicates the motion of loopX owing to the removal of the C-terminal domain. A sodium ion occupying the respective metal-binding site of loopX in L-TYR is thereby lost. (*c*) Magnified superimposition of the respective active sites (A-TYR and L-TYR). (*d*) Superimposition of L-TYR *ab*PPO4 with A-TYR *ab*PPO3. (*e*) Magnified superimposition of the respective active sites (L-TYR *ab*PPO4 and A-TYR *ab*PPO3) depicting a similar location of the placeholder Phe454 and the inhibitor tropolone. (*f*) Superimposed POM-binding region of L-TYR *ab*PPO4 and *ab*PPO3 showing that no conformational change is induced owing to POM binding. (*g*) Superimposition of L-TYR *ab*PPO4 with *ao*TYR. The two loops putatively pushed sideways owing to the attachment of the C-terminal domain of *ao*TYR are indicated as loopX and loopY, respectively. The insert shows the superimposed proteolytic cleavage site located on the back side in the respective perspective of the main view. In contrast to an α-helical location in *ao*TYR, the site is located on a loop in *ab*PPO4. (*h*) Parallel arranged C-terminal subunits of L-TYR *ab*PPO4 (left) and *ao*TYR (right) for a proper comparison. The dashed line indicates missing residues. (*i*) Magnified superimposition of the respective active sites (L-TYR *ab*PPO4 and *ao*TYR).

The A-TYR and L-TYR chains were superimposed to analyze structural changes induced by the proteolytic removal of the C-terminal domain, which shields the active site (Fig. 4 ▶ *a*). However, only small structural changes in the main core were perceivable [root-mean-square deviation on C^α^ atoms (r.m.s.d.^Cα^) = 0.230 Å; overall root-mean-square deviation (r.m.s.d.^total^) = 0.280 Å] beside a marked change in the location of the loop region between α8 and α9 (loopX; Asn236–Ser246; Fig. 4 ▶ *b*). In L-TYR, this loop region occupies a position sideways towards the ‘flat bottom’ side of the conical main core, which is flipped orthogonally in the A-TYR chain by the hypothetical removal of the C-terminal domain. Interestingly, a metal binding site (Na2B) is lost owing to this side turn. This loop is followed by the α9 helix, which contains two copper-coordinating histidines (His251 and His255); however, almost no conformational change is observed. Only a minor alteration of the side-chain posture is discernible at the two copper-binding histidine positions (Fig. 4 ▶ *c*). Following the α9 helix, another loop (Gly259–His266) and a small 3_10_-helix (η2) turn into the α10 helix in the antiparallel direction. The α10 helix is host to the HH motif (His282 and His283), which is conserved in tyrosinases and in which His283 coordinates the copper B ion (CuB; Fig. 4 ▶ *c*). Apart from a different side-chain orientation (Arg260) and a side-chain flip of His282, no significant conformational change is apparent. Such a change might normally be expected in this region (α9 and α10) owing to the proteolytic removal of the C-terminal domain, which could explain the observed copper flexibility (outlined below) of the CuB site of A-TYR.

### 3.3. Active site   

**Figure 5:**
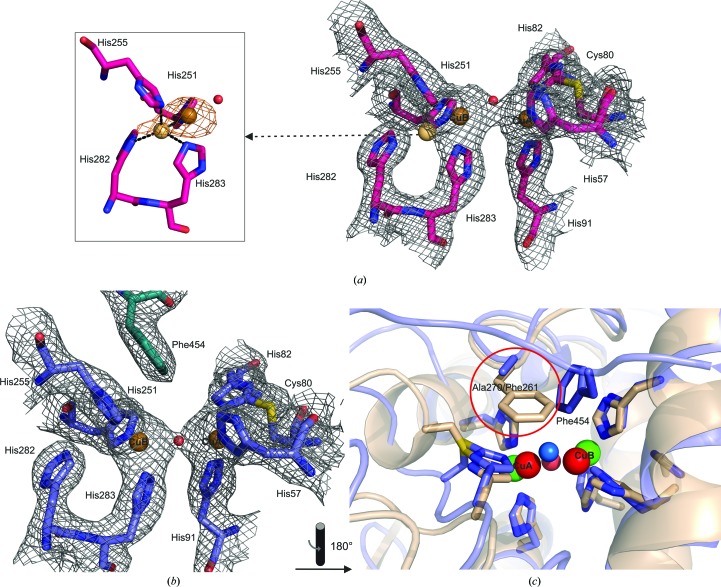
Active site of L-TYR and A-TYR *ab*PPO4. Colour code: A-TYR, purple; L-TYR, blue; copper ion, bronze (alternative conformation, light bronze); 2*mF*_o_ − *mF*_c_ electron-density mesh (0.63 e Å^−3^, 1.5 r.m.s.d.), grey; anomalous electron-density mesh (0.40 e Å^−3^, 3 r.m.s.d.), orange; catechol oxidase from *I. batatas*, beige; L-TYR *ab*PPO4 copper ions, green; respective superimposed *ib*CO copper ions, red. (*a*) Active site of A-TYR lacking the protruding Phe454 (C-terminal domain) with a copper distance of 4.2 Å. A water molecule (Wat128, ocupancy 0.90, *B* = 42.65 Å^2^) is bridging the two copper ions in a triangular position (CuA–Wat = 2.4 Å; Wat–CuB = 2.3 Å). CuB shows an alternative conformation in the vicinity of His282 (CuB1–CuB2 = 2.3 Å). His255, His251, His282 and His283 are coordinating the weakly occupied alternative CuB site (CuB1, occupancy 0.92, *B* = 30.62 Å^2^; CuB2, occupancy 0.08, *B* = 29.31 Å^2^) in a tetrahedral conformation (Cu—N^∊/δ^ = 2.1–2.2 Å). The inset shows the anomalous scattering electron-density map, which clearly exhibits a hump towards His282. Thus, positional flexibility of the CuB site is indicated. Notably, His282 in (*a*) is side-chain flipped compared with (*b*). (*b*) Latent active site of L-TYR showing defined electron density at both copper-binding sites. A water molecule (Wat129, occupancy 0.95, *B* = 32.90 Å^2^) is bridging the copper (CuA–CuB = 4.7 Å) spheres positioned directly on a line between the two ions (CuA–Wat = 2.3 Å; Wat–CuB = 2.3 Å). (*c*) Superimposition of the active site of L-TYR *ab*PPO4 and catechol oxidase from *I. batatas*. The CuA site-blocking bulky residue (Phe261) in *ib*CO is substituted by the less bulky alanine residue (Ala270) in L-TYR *ab*PPO4.

The active site of L-TYR exhibits the deoxy-state conformation with a Cu^I^–Cu^I^ distance of 4.7 Å, and all copper-coordinating histidines (N^∊2^) are found at the proper remote distances of around 2 Å (Fig. 5 ▶ *b*). A water molecule is positioned on a line between the two coppers. The CuA–CuB distance of 4.2 Å in the A-TYR chain is slightly shorter and the water molecule (occupancy 0.9) is also present but in a triangular position to the two coppers (Fig. 5 ▶ *a*). The active site of A-TYR therefore appears to be in the met state, which is defined as the resting state where the two cupric (CuII) ions are bridged *via* a hydroxide or a water ligand at a distance of approximate 3.4 Å (Solomon *et al.*, 1996 ▶). However, as shown previously for a catechol oxidase from grapes (PDB entry 2p3x; Virador *et al.*, 2009 ▶), the two coppers here may adopt rather distant (4.2 Å) positions from each other.

Nonetheless, one distinct residue (Phe454) originating from the loop region right after β11 is clearly protruding towards the CuA site of L-TYR (Fig. 5 ▶ *b*). This phenylalanine residue is supposed to act as a placeholder for a potential substrate, as described recently (Fujieda *et al.*, 2013 ▶). This has similarly been described previously for a tyrosine residue of the attached caddie protein (ORF378) from *S. castaneoglobi­sporus* tyrosinase (*sc*TYR) as well as for a phenylalanine residue from *M. sexta* pro-tyrosinase and several arthropod haemocyanins (Hazes *et al.*, 1993 ▶; Matoba *et al.*, 2006 ▶; Li *et al.*, 2009 ▶). Therefore, an active-site blocking function of the C-terminal domain in a comparable manner can be supported by the data presented here.

In catechol oxidase structures a bulky residue (mostly Phe) positioned atop the CuA site has been described to be responsible for hindering the monophenolase activity (Klabunde *et al.*, 1998 ▶; Matoba *et al.*, 2006 ▶). A superimposition of the active sites of *ib*CO and L-TYR *ab*PPO4 (Fig. 5 ▶ *c*) shows that an alanine residue is located at this position in *ab*PPO4, which endorses the aforementioned assumption.

The C^∊1^ atom of His81 and the S^γ^ atom of Cys79 exhibit a covalent thioether bond in both chains. This exceptional post-translational modification is common in eukaryotic type-3 copper centres (Gielens *et al.*, 1997 ▶). In a recent study of *ao*TYR, a mechanism for the formation of this thioether bridge owing to the incorporation of copper ions was depicted (Fujieda *et al.*, 2013 ▶).

The crystal structure of *ab*PPO4 described here with a crystallographic heterodimeric constitution allows a direct comparison between the two active sites of a latent (zymogen) tyrosinase (L-TYR) and its proteolytically activated form (A-TYR). In Fig. 4 ▶(*c*), a superimposition of the respective active sites is presented.

All three of the histidines coordinating copper A (CuA; His57, His82 and His91), the covalent thioether bridge linking Cys80 to His82, and the CuA sphere superimposed well. Hence, the CuA site seems to be fixed irrespective of whether or not the C-terminal domain is present. However, the CuB-coordinating histidine side chains (His251, His255 and His283) as well as the CuB ion have only slightly shifted conformations. Interestingly, positive electron density (*mF*_o_ − *mF*_c_) is observed between CuB and His282 (HH motif) at a distance of 2.3 Å from CuB. Thus, an alternative conformation was assumed for the position of CuB ion in which it is coordinated in a trigonal-pyramidal fashion (nearly tetrahedrally) by four histidine ligands (His251, His255, His282 and His283). His282 is a residue originating from the α10 helix (Figs. 2 ▶, 4 ▶ *c* and 5 ▶ *a*) and is conserved throughout tyrosinases. Thus, a coordination of the CuB ion that includes this seventh His282 may be assumed to be an alternative conformation with a minor probability (CuB2; occupancy 0.09). This would result in a somewhat flexible CuB site. In the inset in Fig. 5 ▶(*a*) the anomalous electron density for the CuB site is shown. Here, a hump of the electron-density sphere around CuB is clearly expanding towards His282, indicating that the density is not caused by water but by an anomalous scatterer. Notably, a similar situation for copper flexibility in tyrosinases was observed for the CuA site in *sc*TYR (Matoba *et al.*, 2006 ▶). However, in this case no additional histidine is involved in cooper coordination but one of the involved histidines shows an alternative conformation, resulting in a second binding mode for CuA. Notably, in *sc*TYR no thio­ether bridge was found for the CuA site.

The observed copper flexibility for the CuB site could contribute to elucidation of the tyrosinase mechanism, particularly the monophenolase activity, which is still not fully understood.

### 3.4. Proteolytic cleavage site   

The latent precursor enzyme (L-TYR) is transformed to its active form (A-TYR) by removal of the C-terminal domain (Faccio *et al.*, 2013 ▶; Fujieda *et al.*, 2013 ▶). The proteolytic cleavage site (post-Ser383), which was precisely determined by mass spectrometry, is located on a loop (Ile380–Asn388) protruding into the surrounding solvent which follows the common tyrosinase YG motif (Mauracher, Molitor, Michael *et al.*, 2014 ▶; Figs. 1 ▶ *c*, 2 ▶ and 4 ▶ *g*, inset). This accessible location can be easily approached by a protease of a still unknown origin and type. Similar to *ao*TYR, a hydrogen-bond interaction between Tyr279 of the conserved YG motif and Arg288 as well as Asp133 of the main core is present in both forms (A-TYR and L-TYR), thus forcing this region to associate with the main core. However, in contrast to the *ao*TYR crystal structure, the cleavage site is not located on a π-helix but on a loop (Fig. 4 ▶ *g*, inset). Notably, in contrast to the A-TYR *ab*PPO3, which ends at the YG motif (in the crystal structure), the A-TYR *ab*PPO4 chain continues for an additional four amino acids to Ser383.

### 3.5. C-terminal domain   

The C-terminal domain consisting of 182 residues (Glu384–Thr565) contains four α-helices, one 3_10_-helix and ten β-strands and is attached to the ‘flat bottom’ side of the main core in such a way that the active site is shielded (Fig. 1 ▶ *d*). Curiously, the electron density assigned to the C-terminal domain (Glu384–Thr558) of the enzyme was solely located in the *B* chain. The electron density for all expected residues was found except for the last seven C-terminal residues (Val559–Thr565). Their absence might be explained by a disordered arrangement. Six long β-strands (β9–β13 and β15) and two short β-strands (β17 and β18) form a β-barrel structure (Fig. 1 ▶ *c*). Crucial for the C-terminal shielding function is the region Ile448–Thr455 (starting with β11), which protrudes through the solvent-accessible groove of the active site (Fig. 1 ▶ *d*). The residue Phe454 acts as the substrate placeholder, pointing directly towards CuB at a distance of 4.3 Å (C^∊2^–CuB). Interestingly, the following range of residues (Arg446–Leu471) forms a relatively long loop extending from the main core of the protein. This loop bends with a small angle halfway and integrates again into β12. The conserved tyrosinase C*XX*C motif of the protein (Cys462–Cys465), which is also a crucial motif in copper chaperones (Davis & O’Halloran, 2008 ▶; Robinson & Winge, 2010 ▶; Figs. 1 ▶ *c* and 1 ▶ *d*), is located at its turnover. No disulfide-bonded state of this motif is found, in contrast to the previously reported MS measurements (Mauracher, Molitor, Michael *et al.*, 2014 ▶). This motif has been described to be responsible for the incorporation of copper ions into the active site. The proposed mechanism does work in a non-disulfide-bonded state (Fujieda *et al.*, 2013 ▶). One can speculate that this part of the C-terminal domain acts as a grappler to supply the active site with copper ions.

A similar β-barrel shape was observed for the related structures of *ao*TYR, the light subunit (UniProt G1K3P4) of *ab*PPO3 (Ismaya *et al.*, 2011 ▶; Fujieda *et al.*, 2013 ▶) and the caddie protein in the *sc*TRY crystal structure (Matoba *et al.*, 2006 ▶). Only low sequence identities and structural similarities to these light subunits are found for the C-terminal domain of *ab*PPO4 (*ao*TYR, sequence identity 16%, r.m.s.d.^Cα^ = 4.3 Å; *ab*PPO3 light subunit, sequence identity 12%, r.m.s.d.^Cα^ = 14.6 Å; *sc*TYR *caddie*, sequence identity 8%, r.m.s.d.^Cα^ = 11.3 Å). Apart from the active-site blocking and copper-incorporation function, no further purposes of this peculiar domain, which constitutes one third of the whole protein, are known. A structural homology search for the C-terminal domain using the *DALI* server (Holm & Rosenström, 2010 ▶) gave structural similarities to lipoxygenases (PDB entries 2p0m and 1lox) and lectins (PDB entries 1ous and 2xr4) in addition to hits for obviously related proteins [*ao*TYR (PDB entry 3w6w) and haemocyanin (PDB entry 1js8)]. Interestingly, lipoxy­genases are similarly constituted to tyrosinase precursors (Oliw, 2002 ▶), containing a large ‘nonhaem iron centre’ active-site domain and a smaller N-terminal β-barrel-shaped domain (Oliw, 2002 ▶). The β-barrel domains exhibit a Ca^2+^-stimulated membrane-targeting function (Oldham *et al.*, 2005 ▶). The full-length *ab*PPO4 is known to possess a putative transmembrane anchor in the proteolytically removed C-tail region Ala569–Ala591 (Mauracher, Molitor, Michael *et al.*, 2014 ▶; Fig. 2 ▶). Hence, a membrane-targeting function would be conceivable. Owing to the high similarity of the main core of the tyrosinases (*ab*PPO3, *ab*PPO4 and *ao*TYR), which preserves the functionality of the enzymes, the contrasting lower similarity of the C-terminal domain might explain a subcellular targeting function that differs between the isoforms. With regard to the structural similarity to lectins as indicated by *DALI*, it has to be outlined not only that the light subunit attached to *ab*PPO3 has been described as possessing a lectin-like fold, but also that *A. bisporus* lectin (ABL) was copurified with active mushroom tyrosinase (Rescigno *et al.*, 2007 ▶; Flurkey *et al.*, 2008 ▶; Mauracher, Molitor, Michael *et al.*, 2014 ▶). However, neither a membrane-targeting function nor a carbohydrate-binding function can be shown at this stage, but are indicated as conceivable additional purposes of the C-terminal domain.

### 3.6. Superimposition of L-TYR *ab*PPO4 with A-TYR *ab*PPO3   

In Fig. 4 ▶(*b*), a superimposition of L-TYR *ab*PPO4 with A-TYR *ab*PPO3 (PDB entry 2y9x) is shown. Apart from the loop regions (Gly139–Gly149, Ala72–Cys80 and Leu327–Val332), the two main core structures match very well (r.m.s.d.^Cα^ = 0.681 Å, r.m.s.d.^total^ = 0.709 Å). *Ab*PPO3 was crystallized as a tetramer of two heavy subunits (*ab*PPO3) and two light subunits (ORF239342; UniProt G1K3P4). The light subunit, for which no biological role is known to date, has a lectin-like folding and is supposed to attach to all six PPOs in *A. bisporus* (Weijn *et al.*, 2013 ▶). Interestingly, the light subunits of *ab*PPO3 could notionally attach to *ab*PPO4 in such a way that the C-terminal domain of *ab*PPO4 fits perfectly in a vicinal position (Fig. 4 ▶ *d*). Thus, a similar attachment of the light subunit to *ab*PPO4 cannot be excluded from a steric perspective. However, there is no evidence that such a quaternary structure would exist, since no light subunit was copurified or detected at any stage during the purification procedure (Mauracher, Molitor, Michael *et al.*, 2014 ▶).

The two copper sites of A-TYR *ab*PPO4 and *ab*PPO3 superimpose very well in position and coordination geometry (Fig. 4 ▶ *e*). The copper distance in *ab*PPO3, at 4.49 Å (PDB entry 2y9w; 4.1–4.4 Å for the tropolone-soaked structure, PDB entry 2y9x), is comparable to that of A-TYR *ab*PPO4 (4.19 Å). Interestingly, the cocrystallized inhibitor molecule, tropolone, matches perfectly with the placeholder residue (Phe454) of the C-terminal domain (Fig. 4 ▶ *e*).

### 3.7. Superimposition of L-TYR *ab*PPO4 with L-TYR *ao*TYR (UniProt B8NJ95)   

Although *ao*TYR has a similar total number of amino acids (616) to *ab*PPO4 (611), the main core consists of 458 amino acids and is significantly larger than A-TYR *ab*PPO4 with 383 amino acids. However, even without the missing putative membrane-orientated C-tail (Ala566–Phe611), the C-terminal domain of L-TYR *ab*PPO4, with 188 (of a total of 228) amino acids, is significantly larger than the C-terminal domain of *ao*TYR with 158 amino acids. Notably, in the crystal structure of L-TYR *ab*PPO4 some C-terminal residues have no electron density (Val558–Thr565) but were confirmed to be present by MS measurements (Mauracher, Molitor, Michael *et al.*, 2014 ▶).

Apart from some small α-helices and loop regions, it is possible to superimpose the main cores of the homologous proteins very well (Fig. 4 ▶ *g*; r.m.s.d.^Cα^ = 1.540 Å; r.m.s.d.^total^ = 1.411 Å). Interestingly, the two loop regions (loopX and loopY; L-TYR *ab*PPO4, Asn236–Ser246 and Gly259–His266; *ao*TYR, Ser299–Asn323 and Gly337–His355) are located before α9 and α10, respectively, representing the α-helices where the CuB-coordinating histidines are located, and show similar ‘pushed-aside’ positions owing to the attachment of the C-terminal domain (Figs. 4 ▶ *g* and 4 ▶ *h*). As indicated for L-TYR *ab*PPO4 (for loopX), the motion of those loops might have an influence on a conformational change in the active-site region.

The distance between the two copper ions (3.6 Å) in *ao*TYR is smaller than in *ab*PPO4 (A-TYR, 4.2 Å; L-TYR, 4.7 Å). While the full-length *ao*TYR is described as being in the met state, the L-TYR PPO4 is in the deoxy state (Fig. 4 ▶ *i*).

The C-terminal domains of both homologous enzymes constitute a seven-β-strand barrel fold. However, following the primary chain, the secondary-structure elements and the turnover regions differ more significantly in position and length in comparison to the main core (Fig. 4 ▶ *h*). The conserved C*XX*C motif, described as being involved in copper incorporation into the active site and lacking some electron density in the *ao*TYR structure, is also located on a loop that appears to be disordered (Arg454–Val472) and protrudes from the C-terminal domain in *ab*PPO4 (Fig. 4 ▶ *g*).

### 3.8. POM binding sites   

**Figure 6:**
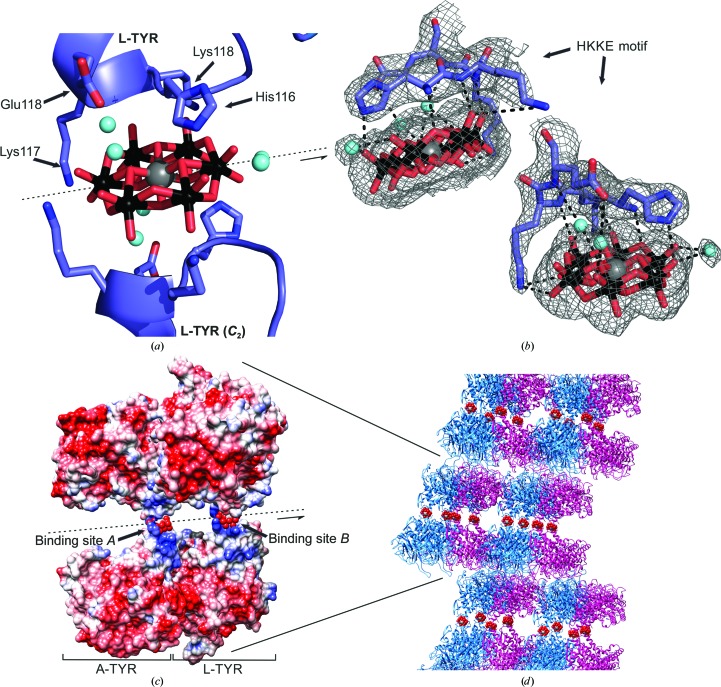
POM (TEW) binding site. Colour code: L-TYR, blue; A-TYR, purple; O atoms, red; Te atom, grey, W atoms, black; water molecules, cyan; hydrogen bonds, dashed black lines; positively charged coulombic surface, blue; negatively charged coulombic surface, red. (*a*) POM-binding (L-TYR) site *B* is located at the tip (start of α-helix α5) of the conical-shaped main core. The polyoxoanion lies on a twofold axis (*C*_2_) embedded by two opposing HKKE motifs (L-TYR and L-TYR *C*_2_). (*b*) Image of two different views of the one-sided POM-binding site *B* with the respective electron density indicated by a grey mesh (2*mF*_o_ −*mF*_c_, 0.40 e Å^−3^, 1 r.m.s.d.) and all possible hydrogen bonds outlined. (*c*) Coulombic surface image of two opposing asymmetric units (heterodimer, A-TYR/L-TYR *ab*PPO4) sharing two polyoxoanions on a twofold axis. This image illustrates how the POM acts as a conjunction linking two otherwise repulsive surfaces. (*c*) Image section of crystal packing displaying a sandwich-like constitution (protein layer–POM layer–protein layer).

In Fig. 6 ▶(*c*), the two POM binding sites in A-TYR *ab*PPO4 and L-TYR *ab*PPO4 are shown. In both cases, the HKKE motif is located at the beginning of the α5 helix right after a turnover from helix α4 and acts like a claw grabbing the disc-shaped polyoxoanion (TEW; Fig. 6 ▶ *a*). At each POM binding site, one of these HKKE claws from the oppositely located asymmetric unit shares one polyoxoanion. Thus, each polyoxoanion is bound by a total of eight amino acids from two equivalent monomers (A-TYR *ab*PPO4 or L-TYR *ab*PPO4). Both polyoxoanions are positioned on a twofold axis by which two opposing asymmetric units, each providing one HKKE motif, are projected on each other (Fig. 6 ▶ *a*). Therefore, it should be outlined that one asymmetric unit contains one heterodimer (A-TYR–L-TYR) and two halves of the non­chiral polyoxo­anion (Fig. 6 ▶ *c*).

For POM binding site *A* (A-TYR) the electron density is less defined compared with POM binding site *B* (L-TYR). In the latter, the POM is very distinctly located on the twofold axis directly between the two HKKE claws (Fig. 6 ▶ *b*). Binding site *A* exhibits a broader electron density perpendicular to the twofold axis. The electron density would offer space for two polyoxoanions that are closely stacked together. However, such a constitution is not possible owing to electrostatic repulsion. Therefore, an alternative position for this polyoxoanion that is not resolved by the electron density is assumed. We refer in the following to POM binding site *B* since the electron density allows a more precise characterization of the binding situation (Fig. 6 ▶ *b*).

Owing to the crystallization conditions at a pH value of 7.5, the lysines involved in POM binding are protonated and hence a hydrogen bond between a lysine N atom and a POM O atom is assumed. The distances of 2.7 Å (N^ζ^–O22 TEW) and 3.19 Å (N^ζ^–O23 TEW) for the laterally positioned Lys118 support this assumption. Lys117 is pointing away from the polyoxoanion (4.8 Å for N^ζ^–O16 TEW); however, only weak electron density for the determination of its actual location is present. In contrast to the lysines involved, His116 is located atop the polyoxoanion with an N^∊^ position that is protonated at the given pH. However, a coordinative interaction between the side chain of His116 and the tellurium of the polyoxoanion is not indicated owing to its too distant positioning (5.3 Å) (Fig. 6 ▶ *b*). Distances of 2.7 Å from N^∊ ^to O8 of the POM are favourable for a hydrogen-bonding interaction. Furthermore, a water-bridged interaction between O^∊^ of Glu119 over Wat12 (3.4 Å) to O12 (2.2 Å) and O18 (2.4 Å) is visible (Fig. 6 ▶ *b*). In addition to these side-chain interactions, the peptide-bond N atoms (His116, Lys117 and Lys118) are at suitable distances (2.7, 3.0 and 2.8 Å, respectively) to be involved in POM binding (Fig. 6 ▶ *b*). No covalent bonds between the POM and the protein were observed as were described for the structure of NTPDase1 cocrystallized with POM (PDB entry 4bvp; Zebisch *et al.*, 2014 ▶).

The detailed characterization assigned to the POM binding mode is appropriate owing to the fact that POM binding site *B* has well defined electron density and a rather low *B* factor (*B* ^TEW_*A*^ = 61.8 Å^2^, *B* ^TEW_*B*^ = 56.1 Å^2^; Fig. 6 ▶ *b*). However, an alternative characterization of the POM binding mode might be described as an electrostatic interaction of a highly negatively charged anion embedded between two oppositely located positively charged protein regions, resulting in a stable interface. In coulombic surface structures of two asymmetric units shown in Fig. 6 ▶(*c*), it is clearly visible that both polyoxoanions bind to regions of the protein with a positive electrostatic potential. Moreover, the POM, owing to its negative charge, connects otherwise repulsive surfaces. This feature comprising a high negative charge on a large rigid molecule makes the TEW polyoxoanion a very suitable agent for the crystallization of otherwise very difficult to crystallize proteins.

In the crystal-packing situation, shown in Figs. 3 ▶ and 6 ▶(*d*), it is illustrated how the POM forms a layer between two protein layers, resulting in a sandwich-like formation. Therefore, the situation might be pictured as follows: if a protein forms a stable crystallographic interface on equivalent monomeric sides but therefore exposes repulsive charged surfaces on the other sides, this would result in impossible interface formation for crystal packing. Owing to its unique structure and high negative charge, a polyoxoanion such as [TeW_6_O_24_]^6−^ could act as a ‘glue’ to connect these otherwise electrostatically repulsive surfaces.

Using a large compound in a high charge state as a cocrystallization agent, one can query whether nonphysiological structural changes are implied in a set of respective crystal structures. In the case of the structure described here, we can entirely disprove a conformational change in the structure, in contrast to the POM-induced domain-orientation change in the structure of NTPDase1 cocrystallized with POM (Zebisch *et al.*, 2014 ▶). This is owing to the mere fact that the respective POM binding domain (α4 and α5 helices) superimposes very well with the comparable structure of mushroom tyrosinase isoform *ab*PPO3 (Ismaya *et al.*, 2011 ▶; Fig. 4 ▶ *f*).

## Supplementary Material

PDB reference: *ab*PPO4 mushroom tyrosinase, 4oua

## Figures and Tables

**Table 1:** Data collection and processing

Diffraction source	MX beamline I04-1, DLS
Wavelength (Å)	0.9173
Temperature (K)	100
Detector	PILATUS 2M
Crystal-to-detector distance (mm)	300.9
Rotation range per image (°)	0.5
Total rotation range (°)	180
Exposure time per image (s)	0.5
Space group	*C*2
*a*, *b*, *c* (Å)	213.53, 83.72, 66.95
α, β, γ (°)	90, 102.53, 90
Mosaicity (°)	0.278
Resolution range (Å)	48.13–2.763 (2.862–2.763)
Total No. of reflections	112311 (10224)
No. of unique reflections	28294 (2621)
Completeness (%)	95.22 (88.86)
Multiplicity	4.0 (3.9)
〈*I*/σ(*I*)〉	8.98 (2.11)
*R*_r.i.m._	0.183
Overall *B* factor from Wilson plot (Å^2^)	45.01

**Table 2:** Structure solution and refinement Values in parentheses are for the outer shell.

Resolution range (Å)	48.13–2.763 (2.862–2.763)
Completeness (%)	95.2
No. of reflections, working set	26875 (2491)
No. of reflections, test set	1415 (131)
Final *R*_cryst_	0.186 (0.2805)
Final *R*_free_	0.228 (0.3497)
No. of non-H atoms	
Total	7753
Protein	7521
Ligand	103
Solvent	129
R.m.s. deviations	
Bonds (Å)	0.011
Angles (°)	0.56
Average *B* factors (Å^2^)	
Overall	42.90
Protein	42.70
Ligand	62.60
Ramachandran plot	
Most favoured (%)	94
Allowed (%)	0.32
